# CSRefiner: a lightweight framework for fine-tuning cell segmentation models with small datasets

**DOI:** 10.1093/bib/bbaf718

**Published:** 2026-01-13

**Authors:** Can Shi, Yumei Li, Jing Guo, Qiuling Chen, Tingting Cao, Sha Liao, Ao Chen, Mei Li, Ying Zhang

**Affiliations:** State Key Laboratory of Genome and Multi-omics Technologies, BGI Research, Shenzhen, 518083, China; BGI Research, Shenzhen, 518083, China; School of Artificial Intelligence, University of China Academy of Science, Beijing, 100049, China; State Key Laboratory of Genome and Multi-omics Technologies, BGI Research, Shenzhen, 518083, China; BGI Research, Shenzhen, 518083, China; State Key Laboratory of Genome and Multi-omics Technologies, BGI Research, Shenzhen, 518083, China; BGI Research, Shenzhen, 518083, China; State Key Laboratory of Genome and Multi-omics Technologies, BGI Research, Shenzhen, 518083, China; BGI Research, Shenzhen, 518083, China; State Key Laboratory of Genome and Multi-omics Technologies, BGI Research, Shenzhen, 518083, China; BGI Research, Shenzhen, 518083, China; State Key Laboratory of Genome and Multi-omics Technologies, BGI Research, Shenzhen, 518083, China; BGI Research, Shenzhen, 518083, China; State Key Laboratory of Genome and Multi-omics Technologies, BGI Research, Shenzhen, 518083, China; BGI Research, Shenzhen, 518083, China; State Key Laboratory of Genome and Multi-omics Technologies, BGI Research, Shenzhen, 518083, China; BGI Research, Shenzhen, 518083, China; State Key Laboratory of Genome and Multi-omics Technologies, BGI Research, Shenzhen, 518083, China; BGI Research, Shenzhen, 518083, China

**Keywords:** cell segmentation, fine-tuning, spatial omics

## Abstract

Recent advances in spatial omics technologies have enabled transcriptome profiling at subcellular resolution. By performing cell segmentation on nuclear or membrane staining images, researchers can acquire single cell level spatial gene expression data, which in turn enables subsequent biological interpretation. Although deep learning-based segmentation models achieve high overall accuracy, their performance remains suboptimal for whole-tissue analysis, particularly in ensuring consistent segmentation accuracy across diverse cell populations. Existing fine-tuning approaches often require extensive retraining or are tailored to specific model architectures, limiting their adaptability and scalability in practical settings. To address these challenges, we present CSRefiner, a lightweight and efficient fine-tuning framework for precise whole-tissue single-cell spatial expression analysis. Our approach incorporates support for fine-tuning widely used segmentation models in the field of spatial omics, while achieving high accuracy with very limited annotated data. This study demonstrates CSRefiner’s superior performance across various staining types and its compatibility with multiple mainstream models. Combining operational simplicity with robust accuracy, our framework offers a practical solution for real-world spatial transcriptomics applications.

## Introduction

Spatially resolved transcriptomics (SRT) technologies have opened a new door for investigating the cellular microenvironment and cellular heterogeneity in complex tissues [1–5], these technologies were named the “Method of the Year 2020” by *Nature Methods* [6]. Recent advances in spatial omics now enable RNA capture at subcellular resolution, as demonstrated by platforms such as Stereo-seq [7] (0.5 μm), Visium HD [8] (2 μm), and Seq-Scope [9] (0.5–0.8 μm). These high-resolution SRT methods enable researchers to investigate single-cell transcriptomes, unlocking insights into functional, developmental, and disease-related mechanisms of living organisms [1]. To obtain single-cell data, cell segmentation serves as the core step for extracting single nuclear or cell boundaries from cell staining images [10–14]. Those single-cell analysis pipelines in spatial transcriptomics (SRT) typically employ state-of-the-art deep learning models for cell segmentation, such as Cellpose [15–18], StarDist [19], and the U-Net-based cell segmentation models^20^.

**Figure 1:**
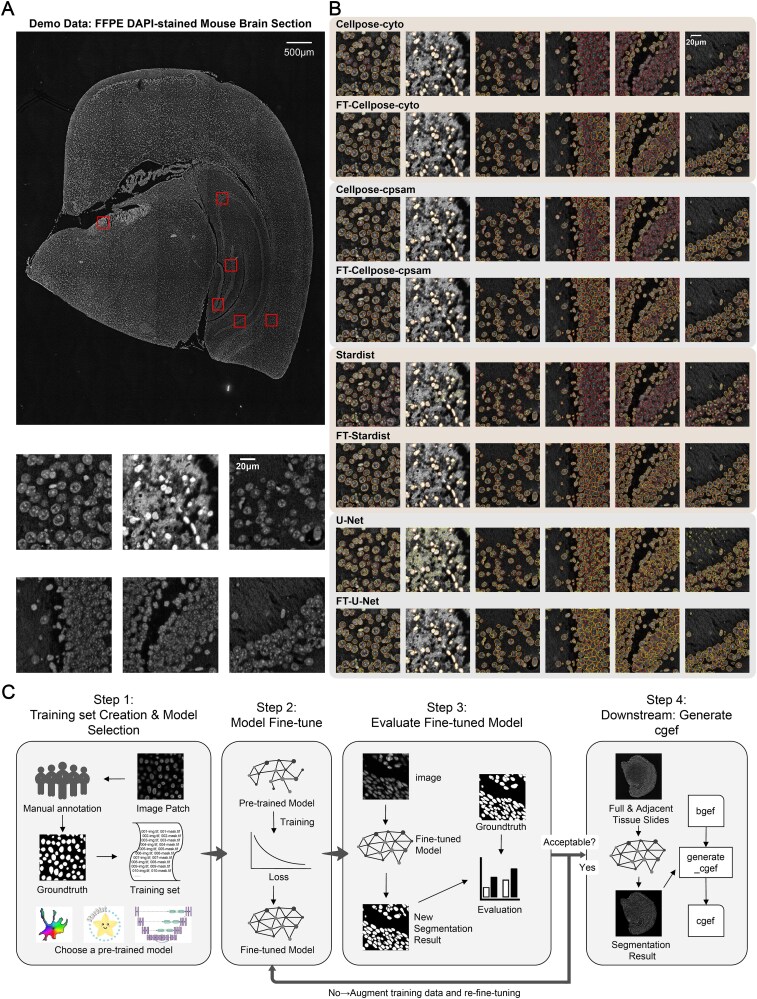
Performance of pre-trained and fine-tuned segmentation models on FFPE DAPI-stained mouse brain slices, and schematic workflow of CSRefiner. (A) FFPE DAPI-stained mouse brain slice images (image resolution: 0.5 μm/pixel) with 6 example regions. (B) Segmentation results of four pre-trained models (Cellpose-cyto, Cellpose-cpsam, StarDist-2D_versatile_fluo, and U-net) and their fine-tuned versions (prefixed with “FT-”, same below) on the 6 example regions, including both hippocampal and non-hippocampal regions. Red outlines denote manually annotated ground truth; yellow outlines denote model-predicted segmentation boundaries. (C) Schematic diagram of the CSRefiner workflow, comprising training set preparation, model selection and fine-tuning, performance evaluation, and subsequent generation of cgef files.

Although current cell segmentation algorithms exhibit satisfactory performance under controlled conditions, their practical application encounters significant challenges due to cellular morphological heterogeneity and variations in image quality [14, 20–22]. These limitations have also been highlighted in recent systematic evaluations of 18 segmentation models across large-scale microscopy datasets, which demonstrate substantial performance variation across tissues, imaging modalities, and cellular morphologies [23]. Specifically in entire sections of complex biological tissues that contain multiple cell types, these algorithms often fail to maintain consistent segmentation accuracy across all cellular populations (see Results and Fig. 1A). Instability across cellular populations and imaging conditions has also been reported in large-scale benchmarking work [24]. Even if the model achieves high segmentation accuracy in the global evaluation, under-segmentation or over-segmentation of local areas may still lead to key segmentation errors, which will further affect the reliability of differential expression analysis, identification of rare cell subpopulations, and construction of cell spatial interaction networks, and even lead to erroneous biological conclusions. Recent fine-tuning frameworks impose key limitations on integrated spatial transcriptomics analysis: first, they are designed around fine-tuning a single proprietary model—constraining flexibility and precluding use of other potentially more suitable architectures; second, they lack a dedicated module to extract single-cell matrices from spatial transcriptomic data. Recent studies have explored parameter-efficient strategies for adapting large pretrained vision models [24, 25], but these approaches are not directly tailored for end-to-end cell segmentation and spatial transcriptomics workflows. In practice, biologists thus rely on manual correction as a last resort, but processing large datasets or multiple samples demands substantial effort (e.g., annotating one 1 cm × 1 cm sample typically takes annotators two weeks or more). This labor-intensive step not only substantially elevates research costs but also severely hinders progress.

In the face of the above challenges, we developed CSRefiner, a lightweight and efficient fine-tuning framework compatible with multiple cell segmentation models, designed to obtain single-cell spatial expression data with accurate whole-tissue segmentation results. By introducing a small sample fine-tuning mechanism, the framework can quickly adapt the existing model to specific image features, thereby significantly improving the segmentation accuracy. Currently, it is compatible with three mainstream cell segmentation models in the field of spatial transcriptomics, namely Cellpose, StarDist, and a U-Net-based cell segmentation model in CellBin—a generalist framework for constructing single-cell expression matrices in spatial omics (hereafter referred to as U-Net), and will be further expandable to support more models in the future. Additionally, it is applicable to a variety of staining methods, such as hematoxylin and eosin (H&E), single-stranded DNA (ssDNA), 4’,6-diamidino-2-phenylindole (DAPI), and multiplex immunofluorescence (mIF). To facilitate evaluation and benchmarking, we also generated and publicly release two demo datasets consisting of formalin-fixed, paraffin-embedded (FFPE) DAPI- and fresh frozen (FF) H&E-stained mouse brain sections with paired expression matrices and annotation results. Collectively, these capabilities enable CSRefiner to significantly minimize manual intervention while preserving segmentation accuracy, particularly in critical regions. This synergistic enhancement not only accelerates the spatial transcriptomics analysis pipeline but also unlocks the potential for robust, large-scale investigations by providing researchers with a scalable and cost-effective annotation strategy.

## Materials and methods

### Tissue samples and data acquisition

#### Experimental animals and ethical approval

Two mouse brain tissue sections were used in this study. Brain tissues were collected from 6-week-old C57BL/6 male mice, provided by the Guangzhou Institutes of Biomedicine and Health, Chinese Academy of Sciences, in collaboration with the BGI laboratory. All animal experiments followed institutional ethical guidelines and were approved by the relevant committees.

#### Tissue preparation and staining

FFPE DAPI-stained mouse brain section: Fresh mouse brains were dissected and fixed in 4% paraformaldehyde at room temperature for 24-48 hours, followed by dehydration, paraffin embedding, and sectioning into 5 μm-thick slices using standard histopathological protocols. Sections were mounted on Stereo-seq Chip N after flattening in a 45°C water bath, then baked at 42°C for 3 hours and dried overnight. Deparaffinization, decrosslinking, and post-decrosslinking fixation were performed according to the STOmics Stereo-seq Transcriptomics Set protocol [26]. DAPI staining was conducted after washing with 0.1×SSC buffer supplemented with RNase inhibitor.

FF H&E-stained mouse brain section: Dissected mouse brains were snap-frozen in dry ice-cooled Tissue-Tek OCT and sectioned into 10 μm-thick coronal slices using a cryostat. Sections were mounted on Stereo-seq Chip N, incubated on a 37°C slide dryer for 5 minutes, and fixed in -20°C methanol for 30 minutes. Adjacent sections on glass slides were stained with H&E using a Solabio G1121 kit following the manufacturer’s instructions, then dehydrated, cleared, and mounted with neutral resin.

### Imaging data acquisition

FFPE DAPI-stained mouse brain section: Fluorescence images were acquired using a STOmics Microscope Go Optical system (Scanner Version 1.2.2, 20×/0.5 NA objective, Ximea Mc124 camera) in epi-fluorescence mode. Image quality was verified via the StereoMap software (imageQC module).

FF H&E-stained mouse brain section: Brightfield images were captured using a Motic PA53 FS6 microscope (PA53Scanner 1.0.0.14, 10×/0.75 NA objective, PA53 FS6 SCAN S5LITE RGB module) in epi-brightfield mode.

### Gene expression matrix generation

FFPE and FF sections on Stereo-seq chips were processed for RNA capture, reverse transcription, and cDNA amplification following the STOmics Stereo-seq Transcriptomics Set protocol [26]: FFPE samples used random primers for total RNA capture and FFPE Decrosslinking Reagent for decrosslinking, while FF samples underwent methanol fixation and short-time permeabilization. Libraries were constructed via cDNA fragmentation and PCR, then sequenced on MGI DNBSEQ-T1 (FFPE) or DNBSEQ-Tx (FF) sequencers.

Raw sequencing data were processed with the SAW pipeline [27]: low-quality reads were filtered, CID sequences were mapped to chip coordinates, and reads were aligned to the mm10 genome (STAR software [28]). UMIs for the same CID and gene were collapsed to eliminate PCR duplicates, and spatial gene expression matrices were generated by integrating gene counts with coordinates.

### Model selection, patch generation, and training set construction

To enable efficient fine-tuning with limited annotated data, CSRefiner recommends constructing a concise and information-rich training set from the target image. The core process is as follows.

First, users need to select a pre-trained model. Apply a pre-trained segmentation model supported by CSRefiner (such as Cellpose, StarDist, and U-Net) to the target tissue image. Visually inspect the initial segmentation results using tools such as ImageJ (evaluating global coverage and local boundary accuracy), and select the model with the best overall performance as the starting point for fine-tuning. If manually annotated ground truth is available, the evaluation module of CSRefiner can also be used to quantitatively evaluate and assist in model selection.

CSRefiner's lightweight cropping tool is then used to batch generate training patches from the whole tissue image. With the exception of the StarDist H&E-optimized pre-trained model (which requires 512×512 pixel patches), all other models use 256×256 pixel patches. The built-in boundary reflection fill tool can be used to expand 256×256 pixel images to 512×512 pixels without altering cell morphology, making them compatible with StarDist and avoiding additional data preparation.

When selecting labeled patches, prioritize coverage of areas with poor segmentation quality (e.g., areas with blurred cell boundaries, dense overlap, or uneven staining leading to under- or over-segmentation). At the same time, appropriately include regions with simple morphology and sparse cells to ensure model generalization (to avoid overrepresentation of these areas and potential model forgetfulness). Labeling can be performed using tools such as QuPath. For reference, based on our experience, manually annotating a single 256×256 image takes approximately 15 minutes. The conversion tool provided by CSRefiner automatically converts the semantic masks generated by QuPath into instance masks (which assign a unique integer identifier to each cell) required by instance segmentation models such as Cellpose and StarDist.

Finally, CSRefiner organizes the annotated images and corresponding masks into a standardized trainset list for subsequent fine-tuning and dataset version tracking. As a practical starting point, preparing around ~300 annotated cells generally provides a reasonable initial fine-tuning effect, but the optimal number depends on tissue complexity. If the initial fine-tuning fails to meet expectations, incrementally add labeled samples and re-fine-tune based on the original pre-trained weights (to avoid catastrophic forgetfulness) rather than using the previously fine-tuned checkpoint.

### Base models and fine-tuning procedures

Cellpose (cyto, cyto3, nuclei, etc.)

Cellpose is a general cell segmentation model based on residual U-Net. Several pre-trained models for various tissue types and staining methods have been officially released (e.g., cyto, cyto3, and nuclei).

CSRefiner integrates an automatic fine-tuning pipeline based on the official Cellpose training API, “cellpose.train.” Users load training data by inputting a trainset list, which supports grouping by sample identifier (SN) and proportionally splitting the training/validation sets. Training is GPU-accelerated by default, using the SGD optimizer (with an initial learning rate of 0.2 and a weight decay of 1e-5). Loss curves are automatically recorded and visualized during training, and output weights and log files are stored in a timestamped directory. Switching between different pre-trained models is supported with one click.

### Cellpose-SAM (cpsam)

Cellpose 4.0 introduces a new architecture that integrates the SAM image encoder with the Cellpose flow prediction mechanism, making it even more suitable for segmentation in dense cell images. The fine-tuning process is consistent with Cellpose (including data loading, SN grouping, and loss tracking), with the only adjustments being a small batch size (batch size = 1) and a low learning rate (1e-5) to ensure training stability, as recommended by the official website. It performs exceptionally well in areas with blurred cell edges and densely packed cells, making it particularly well-suited for fine-tuning segmentation in highly heterogeneous tissue images, such as those found in spatial transcriptomes.

### StarDist

StarDist uses a U-Net backbone network and models target cells as star-convex polygons. CSRefiner addresses the lack of early stopping in the official framework through single-epoch iterative training: validation loss is recorded after each epoch, and training is terminated when the validation loss stabilizes (with a patience of 30). It supports initialization from scratch, official pre-trained weights, or historical checkpoints. It uses single-channel grayscale and percentile intensity normalization for fluorescent images and supports three-channel input for H&E images. Training curves and versioned output files are automatically generated.

### U-net

The U-Net model is based on the BCDU-Net D3 architecture. ConvLSTM units are introduced in the decoder to enhance spatial context modeling, making it suitable for images with complex staining and high background noise. Fine-tuning is implemented in TensorFlow 2.x, with data loaded and augmented via a custom DataGenerator. H&E images are color normalized and processed with CLAHE, while ssDNA/DAPI images are converted to grayscale, percentile-normalized, and adaptively histogram-equalized. The Adam optimizer with binary cross-entropy loss is used, along with ReduceLROnPlateau (dynamic learning rate adjustment) and early stopping (patience = 30) to prevent overfitting. The model supports initialization either from scratch or using pre-trained .hdf5 weights, and all output files are systematically archived to ensure reproducibility.

After fine-tuning, CSRefiner's unified inference interface can be used to perform segmentation on whole tissue images or batches of unannotated patches, generating results that are compatible with downstream spatial transcriptomics analysis pipelines.

### Cgef file generation

cgef is a commonly used format in the spatial transcriptomics field for integrating gene expression matrices with cell segmentation results. CSRefiner relies on the `cgef_writer_cy.generate_cgef` function in the gefpy library to associate the segmentation mask with the original expression matrix (in .gem.gz or .gef format). The principle is to map each expression point to the corresponding cell segmentation region, aggregate the gene counts of all expression points within a single cell mask, and generate a single-cell gene expression matrix with cell centroid coordinates. The resulting .cgef file can be directly used in downstream spatial transcriptomics research, such as cell annotation, clustering, and differential expression analysis.

### Cell segmentation performance evaluation metrics

In order to quantitatively compare the performance of different models and fine-tuning schemes on the cell segmentation task, this study used five classic metrics (Table 1): Precision, Recall, F1 Score, Jaccard Index, and Dice Coefficient, focusing on the overlap and matching quality between the predicted and true masks.

**Table 1:** Cell segmentation performance evaluation metrics.

Metric	Description	Formula (IoU threshold = 0.5)
Precision	Proportion of true cells among predicted positives	$Precision=\frac{TP}{TP+ FP}$
Recall	Proportion of true cells correctly identified	$Recall=\frac{TP}{TP+ FN}$
F1 Score	Harmonic mean of precision and recall	$F1\ Score=2\times \frac{precision\times recall}{precision+ recall}$
Jaccard Index	Ratio of intersection and union between predicted and ground truth masks	$Jaccard=\frac{TP}{TP+ FP+ FN}$
Dice Coefficient	Similar to Jaccard, but more sensitive to small targets	$Dice=\frac{2\times TP}{2\times TP+ FP+ FN\ }$

Note: TP (true positive) = predicted cell correctly matched to a ground-truth cell; FP (false positive) = predicted cell with no corresponding ground-truth cell; FN (false negative) = ground-truth cell not detected by the model. All metrics were computed using an IoU (Intersection over Union) threshold of 0.5 for mask matching.

All metric results are calculated based on the model's predicted masks and the corresponding ground-truth masks, and are recorded in .xlsx files for easy traceability. These metrics are visualized using the Python matplotlib [29] library to intuitively illustrate the differences in average performance and inter-sample stability between different models.

### Quantification of cellular morphological and spatial metrics

This study, based on TIFF-format cell images and corresponding binary segmentation masks, uses a custom CellMetricsCalculator class to automatically extract six core metrics to quantify cell spatial arrangement, density, morphological complexity, and boundary integrity. Image preprocessing and metric calculation were performed using Python [30] with core libraries including scikit-image [31] for image processing, NumPy [32] for numerical computations, pandas [33] for data organization, and SciPy [34] for spatial distance calculations.

The specific process involves first labeling individual cells through connected region analysis to extract basic properties such as cell area, perimeter, and center of mass coordinates. Six metrics are then calculated based on these properties (Table 3): Mean Distance, which reflects cell dispersion; Compactness, which indicates the compactness of cell packing; Density, which reflects cell distribution density; Distribution, which measures distribution heterogeneity; Shape Complexity, which reflects the degree of shape irregularity; and Edge Contrast, which indicates boundary clarity. The calculation logic for each metric focuses on the direct correlation between image features and biological significance, ensuring the interpretability of the results.

### Downstream transcriptomic analysis

To verify the impact of improved segmentation accuracy on biological analysis, this study conducted downstream analysis on the cgef file output by CSRefiner based on the spatial transcriptome analysis platform SDAS (available at https://github.com/STOmics/SDAS). The core workflow is as follows:

First, the cgef file was converted to the AnnData format using the Stereopy [35, 36] tool. Cell type annotation, differentially expressed gene (DEG) analysis, and functional enrichment analysis were then performed sequentially. Cell type annotation was performed using the cell2location [37] module of SDAS. Each cell was assigned to the reference cell type with the highest posterior probability. The annotation confidence score represents the posterior probability, indicating the reliability of the assignment; higher scores correspond to greater confidence. Changes in the distribution of annotation scores before and after fine-tuning were analyzed. DEG analysis screened for significant DEGs (FDR < 0.05 and |log₂ fold change| > 1) for each annotated cell type using the Wilcoxon rank sum test, focusing on differences in the number of DEGs among cell populations in the hippocampus. Functional enrichment analysis was performed based on the GO biological process (2023) [38, 39] annotations of Enrichr [40], and up-regulated and down-regulated DEGs were identified. Enrichment analysis identified pathways overrepresented among DEGs, with the Rich Factor defined as the ratio of DEGs annotated to a given pathway to the total number of genes annotated to that pathway, reflecting the relative representation of significant genes. Key pathways were visualized using bubble and waterfall plots, highlighting biologically meaningful processes. Post-fine-tuning enrichment results were compared to pre-fine-tuning results to assess improvements in the biological relevance of spatial transcriptomic data.

## Results

### CSRefiner framework overview

Before developing CSRefiner, we first evaluated the four segmentation models supported by CSRefiner for fine-tuning on three representative spatial transcriptomics datasets: FFPE DAPI-stained mouse brain sections, FF H&E-stained mouse brain sections, and FF H&E-stained mouse lung sections (Fig. 1A, Supplementary Fig. S1A, Supplementary Fig. S2A). Visual inspection of segmentation results (Fig. 1B, Supplementary Fig. S1B, Supplementary Fig. S2B) revealed noticeable performance inconsistencies. In regions with sparse cells and clear nuclear boundaries, the models generally produced reasonable segmentations; however, in morphologically complex regions—such as the hippocampus in brain sections or areas with densely packed alveolar structures in lung tissue—pre-trained models exhibited pronounced errors.

This inability of mainstream models for SRT to maintain visually reliable segmentation in complex tissue regions (e.g., the hippocampus) motivated us to develop CSRefiner. As illustrated in Fig. 1C, CSRefiner is a lightweight and flexible pipeline designed to rapidly adapt pre-trained segmentation models to tissue-specific features in spatial transcriptomics images. The workflow consists of four streamlined steps. First, users construct a small training set by annotating representative patches from regions where baseline segmentation is suboptimal, such as densely packed or morphologically heterogeneous regions (e.g., hippocampus). A base model is then selected from supported models (Cellpose, StarDist, or U-Net). Second, the chosen model is fine-tuned on the annotated patches through a unified, parameter-controlled script. Third, the refined model is evaluated on the training set/testing set and iteratively refined by adding more annotated patches if necessary, ensuring that the model achieves satisfactory performance across challenging regions. Finally, the optimized model is applied to whole-slide images, and the resulting segmentation boundaries are integrated with the corresponding expression matrix to generate a single-cell-level gene expression format (cgef) file, a widely used standard in spatial transcriptomics, facilitating downstream single-cell analyses.

### CSRefiner improves segmentation accuracy with minimal manual effort

#### Improvement of segmentation performance

In non-hippocampal regions, CSRefiner achieves more refined segmentation, closely adhering to actual cell nucleus outlines and rarely missing cells (Fig. 1B). U-Net, which previously frequently produced false positives in the background or over-segmented cells, now generates complete cell outlines, significantly reducing the fragmentation of individual nuclei into disjoint fragments. In the hippocampus, where dense cell packing and blurred nucleus boundaries lead to severe false negatives in pre-trained models, CSRefiner achieves significant improvements: it successfully recovers many previously overlooked cells and can distinguish individual nuclei even in the densest clusters. Although U-Net's boundary delineation accuracy remains slightly lower than Cellpose and StarDist (consistent with its inherent emphasis on recall), its segmentation results are visually much more consistent than the baseline models. Details of the training set and training hyperparameters used in this study are shown in Supplementary Table 1 and 2.

**Figure 2:**
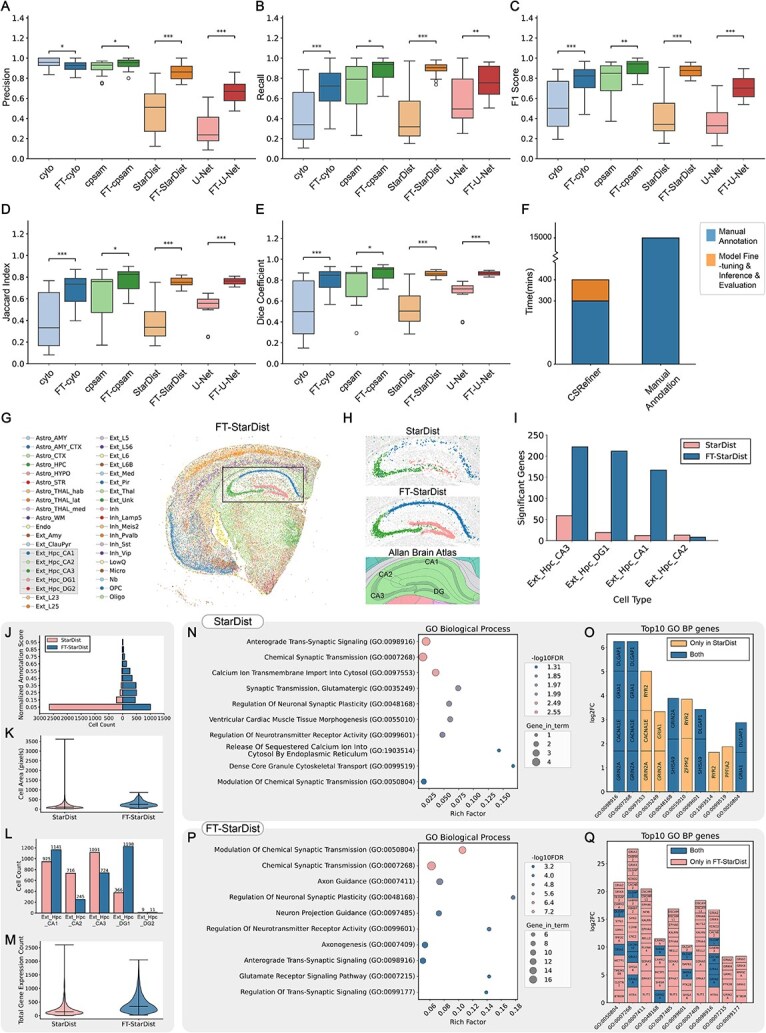
Segmentation performance and downstream biological analyses improved by CSRefiner. (A–E) quantitative evaluation of segmentation performance before and after fine-tuning across four representative models (Cellpose-cyto, Cellpose-cpsam, StarDist, and U-net). Boxplots show improvements in (A) precision, (B) recall, (C) F1 score, (D) Jaccard index, and (E) dice coefficient. Significance lines and P values are shown on the boxplots, with 1–3 asterisks indicating P < 0.05, 0.01, and 0.001, respectively. (F) Time required for manual whole-slide annotation (~10 days) versus CSRefiner-assisted workflow (~400 minutes). (G) Spatial maps of cell type annotations generated by cell2location using cgef matrices from the fine-tuned StarDist model. (H) Visualization of segmented and annotated cells in hippocampal subregions before and after fine-tuning compared with the Allan brain atlas for the hippocampus. (I) Comparison of the number of significant genes in each cell subtype in the hippocampus before and after fine-tuning. (J) Distribution of normalized annotation scores in the hippocampus before and after fine-tuning. (K) Distribution of cell areas in the hippocampus before and after fine-tuning. (L) Comparison of the number of cells of each cell subtype in the hippocampus before and after fine-tuning. (M) the number of genes detected per cell in the hippocampus before and after fine-tuning. (N–Q) gene ontology enrichment analysis results for Ext_Hpc_DG1 before and after fine-tuning. In the bubble plot, bubbles positioned further right indicate stronger enrichment, with deeper red showing higher significance and larger size representing more hit genes. The stacked bar plot displays contributing genes for each GO term, where bar height reflects contribution strength (taller bars highlight potential driver genes). Orange denotes terms/genes present only before fine-tuning, blue indicates both, and pink marks those unique to after fine-tuning.

We next assessed the impact of CSRefiner on segmentation accuracy using five standard evaluation metrics: precision, recall, F1 score, Jaccard index, and Dice coefficient (Fig. 2A–E). Boxplot comparisons showed improvements across almost all metrics for all tested models. Cellpose-cyto F1 increased from 0.54 to 0.78, Cellpose-cpsam from 0.79 to 0.91, StarDist from 0.42 to 0.87, and U-Net from 0.37 to 0.71. Similar gains were observed for precision, recall, Dice, and Jaccard indices. Notably, the improvement was particularly pronounced in models with weaker initial performance, such as StarDist and U-Net, while even the high-performing Cellpose-cpsam model achieved measurable gains. Moreover, the reduced variance in post-fine-tuning scores indicates enhanced consistency and robustness of the models across different image regions. Furthermore, we noted a slight decrease in accuracy for Cellpose-Cyto after fine-tuning. This behavior is expected because the original cyto model showed substantially lower recall due to a large number of missed detections. Fine-tuning effectively reduced these omissions, leading to a notable increase in recall and, consequently, F1 score, at the cost of a marginal reduction in precision. This trade-off reflects improved overall detection completeness. Similar performance improvements were observed on the other two datasets (Supplementary Fig. S1&S2B-G).

#### CSRefiner saves more time than manual annotation

CSRefiner greatly reduced annotation time. We compared the overall time required for CSRefiner-assisted fine-tuning against full manual annotation (Fig. 2F). Based on our empirical annotation experience (~15 minutes per 256 × 256 image patch), generating 20 training patches required ~300 minutes. Model fine-tuning, evaluation, and inference added an additional ~100 minutes, resulting in a total of ~400 minutes (~6.7 hours) for a complete CSRefiner workflow. Table 2 shows the detailed runtime and memory usage of each module. In contrast, manual annotation of an entire whole-slide image would require approximately 10 days (~36 × longer) under comparable conditions. These results demonstrate that CSRefiner enables the generation of high-quality segmentation results within a fraction of the time, significantly lowering the cost and labor of manual annotation.

**Table 2:** CSRefiner module runtime and memory usage.

Model	Task Type	Inference Time	CPU Peak Memory	GPU Peak Memory
Cellpose-cyto	Fine-tuning	32.06s	1.41GB	2.87GB
	Inference (training set)	12.03s	1.27GB	966.00MB
	Inference (full image)	5.91min	42.03GB	17.32GB
Cellpose-cpsam	Fine-tuning	3.54min	1.90GB	4.35GB
	Inference (training set)	14.03s	1.42GB	1.93GB
	Inference (full image)	64.58min	60.24GB	13.43GB
StarDist	Fine-tuning	2.77min	6.85GB	22.39GB
	Inference (training set)	10.05s	3.16GB	22.39GB
	Inference (full image)	3.14min	21.25GB	22.39GB
U-Net	Fine-tuning	5.75min	6.64GB	16.27GB
	Inference (training set)	14.33s	2.89GB	9.36GB
	Inference (full image)	6.08min	44.28GB	5.04GB

Note: Cellpose-cpsam's full image inference runs on a single NVIDIA Tesla T4 GPU (CUDA version: 11.7; 16 GB VRAM). All other experiments run on a single NVIDIA GeForce RTX 3090 GPU (CUDA version: 12.2; 24 GB VRAM). The experimental data included a VisiumHD H&E-stained mouse lung image (full size: 23520×20580 pixels) and a training set of 20 patches (256×256 pixels per patch). “Inference (training set)” refers to processing the 256×256-pixel patches, while “Inference (full image)” refers to the 23520×20580-pixel whole slide. Inference time is defined as the total elapsed time from executing the command to outputting the final results. GPU/CPU peak memory represents the maximum memory usage during the task.

**Table 3:** Cellular morphological and spatial metrics.

Metric	Description	Formula
Mean Distance	Average nearest-neighbor distance	${d}_{ij}=\sqrt{{\left({x}_i-{x}_j\right)}^2+{\left({y}_i-{y}_j\right)}^2}$ ${d}_{\mathit{\min},i}=\mathit{\min}\left({d}_{i1},{d}_{i2},\dots, {d}_{iN},i\ne j\right)$ $Mean\ Distance=\frac{1}{N}{\sum}_{i=1}^N{d}_{\mathit{\min},i}$
Compactness	Normalized nearest-neighbor distance to cell size	$\overline{A}=\frac{1}{N}{\sum}_{i=1}^N{A}_i$ $Compactness=\frac{{\left( Mean\ Distance\right)}^2}{\overline{A}}$ (${A}_i$: Area of cell i, in pixel^2^)
Density	Number of cells per unit area	$Density=\frac{N}{A_{total}}$
Distribution	Coefficient of variation of local densities (higher = uneven distribution, lower = uniform dispersion)	$Distribution=\frac{\sigma_{local}}{\mu_{local}}$ (where local density for cell i: $\frac{3}{D_{i,3}}$, ${D}_{i,k}={\sum}_{i=1}^k{d}_{\mathit{\min},i,j}$)
Shape Complexity	Irregularity of cell shapes (higher = more complex boundaries)	$\overline{P^2}=\frac{1}{N}{\sum}_{i=1}^N{P}_i^2$ $\overline{A}=\frac{1}{N}{\sum}_{i=1}^N{A}_i$ $S\mathrm{h} ape\ Complexity=\frac{\overline{P^2}}{\overline{A}}$ (${P}_i$: Perimeter of cell i)
Edge Contrast	Quantifies contrast between cell edges and background (using Sobel operator)	$Edge\ Contrast=\frac{\mu_{cell}}{\mu_{bg}}$ (${\mu}_{cell}$: Mean intensity of cell edge gradient; ${\mu}_{bg}$: Mean intensity of background gradient)

#### Downstream biological impact of improved segmentation

To further evaluate the biological relevance of CSRefiner, we examined how improved segmentation accuracy influenced downstream spatial transcriptomics analyses.

#### Improved spatial mapping

Using StarDist, the model with the greatest performance gains, fine-tuned segmentation enabled the generation of high-quality cgef matrices. Subsequent cell type annotation with cell2location [37] produced spatial distributions that closely matched established anatomical structures and biological priors (Fig. 2G). Notably, hippocampal subregions were delineated with high fidelity (Fig. 2H), confirming that CSRefiner-generated segmentations directly support biologically meaningful spatial mapping.

#### Improved DEGs detection and annotation quality in the hippocampus

Differential expression analysis of hippocampal cell types revealed that fine-tuning increased the number of significant genes in Ext_Hpc_CA3/DG1/CA1, consistent with visual inspection, indicating improved segmentation of dense hippocampal regions (Fig. 2I).

We then analyzed in detail the changes in cell annotation in the hippocampus before and after fine-tuning. First, annotation confidence scores shifted toward higher values, indicating more reliable cell type assignments (Fig. 2J). Quantitative assessment of cell morphology revealed that extreme cell size outliers were eliminated, while total cell area slightly increased, reflecting more regular and biologically plausible segmentation boundaries (Fig. 2K). For example, before fine-tuning, a few cells showed areas near 3500 pixels while most cells remained below 500 pixels, reflecting segmentation errors. Fine-tuning corrected these anomalies, producing cell size distributions more consistent with expected hippocampal cytoarchitecture. Furthermore, cell type-specific counts exhibited biologically consistent changes (Fig. 2L): Ext_Hpc_CA1 and Ext_Hpc_DG1 cells increased significantly, Ext_Hpc_DG2 remained stable, and Ext_Hpc_CA2/CA3 decreased slightly. These results align with expected patterns of hippocampal cytoarchitecture. Finally, fine-tuning raised the number of detected genes per cell (Fig. 2M), thereby enhancing the sensitivity of downstream transcriptomic analyses. By recovering expression signals from cells that would otherwise be under-segmented or omitted, CSRefiner improved both the resolution and the biological interpretability of spatial transcriptomics data. As shown in Supplementary Fig. S3, similar results were obtained from the analysis of other models.

### Enables deeper biological discoveries

As shown in Fig. 2N–Q, we performed enrichment analysis of Ext_Hpc_DG1 using the GO Biological Process 2023 database [38, 39]. The enrichment analysis quality of the fine-tuned model significantly outperformed that of the pre-trained model. This difference stems from a more accurate cell segmentation mask that ensures the purity of gene expression signals in the cell bin matrix, ultimately outputting enrichment results that are more consistent with the true biological state.

There are significant overlaps in neural/synaptic pathways between the pre- and post-fine-tuning results, such as Chemical Synaptic Transmission (GO:0007268), Modulation of Chemical Synaptic Transmission (GO:0050804), Regulation of Neuronal Synaptic Plasticity (GO:0048168), Regulation of Neurotransmitter Receptor Activity (GO:0099601), and Anterograde Trans-Synaptic Signaling (GO:0098916). Before fine-tuning, this analysis highlighted a limited set of synaptic signaling processes, such as anterograde trans-synaptic signaling (GO:0098916) and chemical synaptic transmission (GO:0007268), supported by a small group of canonical synaptic genes (e.g., GRIA1, GRIN2A, CACNA1E, DLGAP1). Furthermore, the pre-trained top 10 included terms such as Ventricular Cardiac Muscle Tissue Morphogenesis (GO:0055010), which are inconsistent with brain/hippocampal biology, suggesting possible false positive enrichment due to the inclusion of non-target cells/background or noise in the differentially expressed gene list. By contrast, after fine-tuning, the same analysis revealed a broader and biologically coherent network of pathways, including axon guidance, neuron projection guidance, and axonogenesis (Fig. 2P). The expanded gene set (e.g., EPHA4/6/7, SLIT1, DSCAM/DSCAML1, KALRN, CAMK2A/B, GRIA1/2, GRIN2A) is shown in Fig. 2Q and references[41–44]confirm that these genes are indeed implicated in hippocampal neuronal functions. These results suggest that fine-tuned segmentations produce more biologically meaningful gene expression profiles.

### Variable training sample needs for fine-tuning

To evaluate the required training set size for regions of varying segmentation difficulty, we systematically varied the number of annotated cells used for fine-tuning and tested on independent held-out regions not included in the training set.

**Figure 3:**
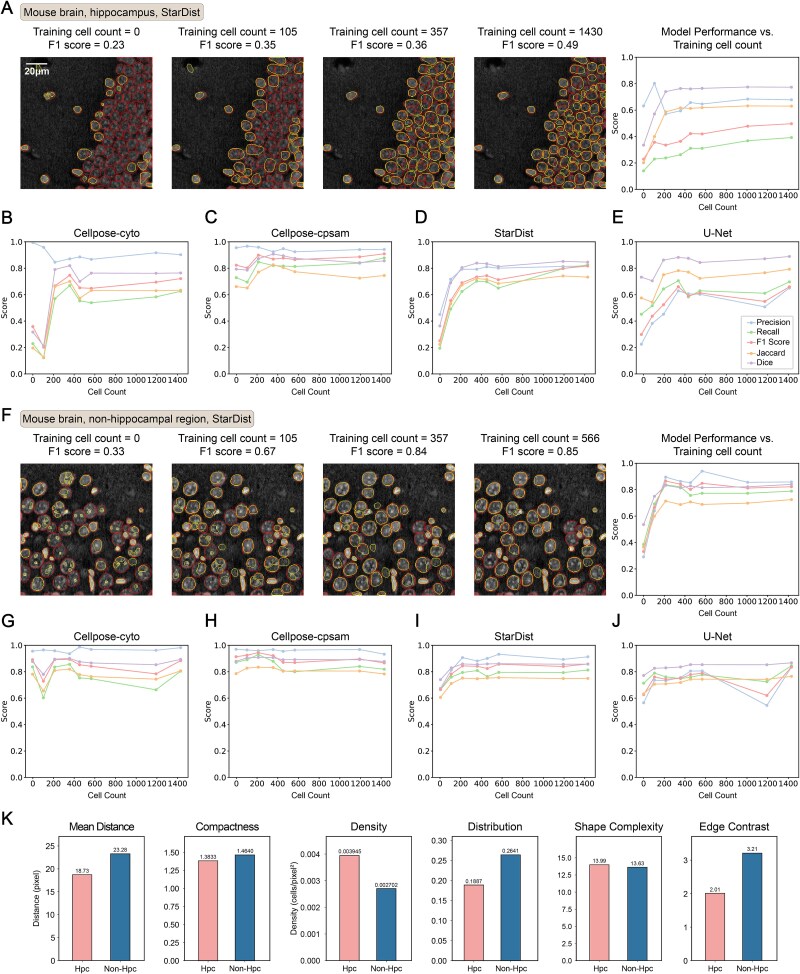
Training set size and tissue complexity influence segmentation performance. (A) StarDist performance on a representative hippocampal test region when fine-tuned with different numbers of annotated cells. The line graph on the right visualizes precision, recall, F1 score, Jaccard index, and dice coefficient for different numbers of training cells. (B–E) visualization of precision, recall, F1 score, Jaccard index, and dice coefficient for Cellpose-cyto, Cellpose-cpsam, StarDist, and U-net across all hippocampal test regions (see table 4) under varying training set sizes. (F) Same as panel (A), evaluated on a representative non-hippocampal test region. (G-J) precision, recall, F1 score, Jaccard index, and dice coefficient for Cellpose-cyto, Cellpose-cpsam, StarDist, and U-net across all non-hippocampal test regions under different training set sizes. (K) Quantitative comparison of regional structural complexity between hippocampal and non-hippocampal images. Metrics include mean distance between cells, compactness, density, distribution, shape complexity, and edge contrast.

As shown in Fig. 3A–E, hippocampal regions required substantially larger training sets. Using StarDist as an example (Fig. 3A), F1 scores increased from 0.23 with no annotated cells (official pre-trained model) to 0.35 with 105 cells, 0.36 with 357 cells, and 0.49 with 1,430 cells. The trend was similar across all four models (Cellpose-cyto, Cellpose-cpsam, StarDist, and U-Net), with performance improving gradually(Fig. 3B–E). These curves indicate that in complex regions, additional annotations continue to provide measurable benefits, though gains become slower as the training set grows and overfitting may eventually occur. In contrast, non-hippocampal regions displayed much milder training requirements. Performance improved sharply with only a small number of annotated cells (Fig. 3F–J). For example, F1 increased from 0.33 (no training cells) to 0.67 (105 cells) and 0.84 (357 cells), and plateaued near 200-300 cells, after which additional annotations yielded diminishing returns. All models showed similar convergence patterns.

To quantitatively assess region-specific complexity, we compared six structural metrics between hippocampal and non-hippocampal images (Fig. 3K). Hippocampal cells exhibited smaller mean inter-cell distance (18.7 vs. 23.3) and lower compactness (1.38 vs. 1.46), indicating tighter packing. Cell density was higher (0.0039 vs. 0.0027), while distribution uniformity was lower (0.189 vs. 0.264), suggesting large-scale dense clustering rather than localized groups. Edge contrast was reduced (2.01 vs. 3.21), reflecting weaker boundaries that complicate segmentation. In contrast, shape complexity was comparable between regions (13.99 vs. 13.63), implying that cell morphology was not a major differentiating factor.

Together, these findings demonstrate that training-set requirements are primarily determined by regional structural complexity. Complex regions benefit from larger training sets, whereas simpler regions require far fewer examples. For new users without prior experience, annotating ~300 cells may serve as a practical initial reference rather than a universal threshold, with the understanding that the optimal number depends on tissue complexity and the chosen model. CSRefiner’s iterative fine-tuning strategy also allows users to expand the training set adaptively if initial performance is insufficient.

## Conclusion and discussion

Accurate cell segmentation is fundamental to spatially resolved transcriptomic analysis, as errors propagate to downstream tasks such as cell type annotation, differential expression analysis, and pathway enrichment. In this study, we present CSRefiner, a flexible framework for fine-tune compatible cell segmentation models. Comparative evaluation demonstrates that CSRefiner improves segmentation accuracy across multiple architectures, reduces annotation time by more than an order of magnitude, and enhances biological interpretability. We also provide three high-quality datasets containing paired images, annotations, and expression matrices as community resources for benchmarking and method development.

Tissue-specific fine-tuning markedly outperforms direct application of pre-trained models, especially in challenging regions such as the hippocampus, recovering missed cells and producing more coherent spatial maps. CSRefiner also demonstrates robustness across preparation protocols, achieving consistent gains on FFPE datasets despite baseline models being trained on FF samples.

Performance scaling analyses revealed that simpler regions saturate with few annotated cells, whereas complex structures require substantially larger training sets. Quantitative tissue metrics—cell density, compactness, and boundary clarity—correlate with segmentation difficulty, providing practical guidance for training set design in large-scale studies.

While CSRefiner reduces annotation requirements compared with full retraining, some manual effort remains necessary, especially in highly complex tissues. In addition, integration with multimodal spatial data, such as joint proteomic profiling, may require further adaptation. Thanks to its modular design, CSRefiner already provides clear interfaces, allowing new segmentation models to be integrated in the future simply by writing an adapter script.

**Key Points**

- CSRefiner is a flexible framework that fine-tunes existing cell segmentation models, substantially improving segmentation accuracy, reducing annotation time, and enhancing biological interpretability.
- Tissue-specific fine-tuning recovers missed cells and produces more coherent spatial maps, even in challenging regions such as the hippocampus.
- CSRefiner demonstrates robustness across preparation protocols, achieving consistent performance gains on FFPE datasets despite baseline models being trained on FF samples.
- Performance scaling analyses links tissue organization metrics (cell density, compactness, boundary clarity) to segmentation difficulty, informing training set design.
- We release three high-quality datasets with paired images, annotations, and expression matrices as community resources for benchmarking and further method development.

## Supplementary Material

Supplementary_Figure1_bbaf718

Supplementary_Figure2_bbaf718

Supplementary_Figure3_bbaf718

Supplementary_Figure_Legend_bbaf718

Supplementary_Table_1_bbaf718

Supplementary_Table_2_bbaf718

## Data Availability

The two mouse brain datasets that support the findings of this study have been deposited into CNGB Sequence Archive (CNSA) of the China National GeneBank DataBase (CNGBdb) with accession number CNP0007731: https://db.cngb.org/search/project/CNP0007731/. The mouse lung dataset generated by the 10x Genomics VisiumHD platform is publicly available at: https://www.10xgenomics.com/datasets/visium-hd-cytassist-gene-expression-mouse-lung-fresh-frozen. Additionally, we have uploaded these datasets to Zenodo: 10.5281/zenodo.17098314.

## Code availability

Project home page: https://github.com/STOmics/CSRefiner.

## Notes

**Biographical note:** The authors are a collaborative research team from BGI Research, Shenzhen, with research interests in spatial omics and cell segmentation, focusing on addressing practical challenges in large-scale biological data analysis.
