# Supplementary figures and images for: CSRefiner: a lightweight framework for fine-tuning cell segmentation models with small datasets
Source: Brief Bioinform. 2026 Jan 13;27(1):bbaf718. doi: 10.1093/bib/bbaf718 (PMC12796817; doi:10.1093/bib/bbaf718)

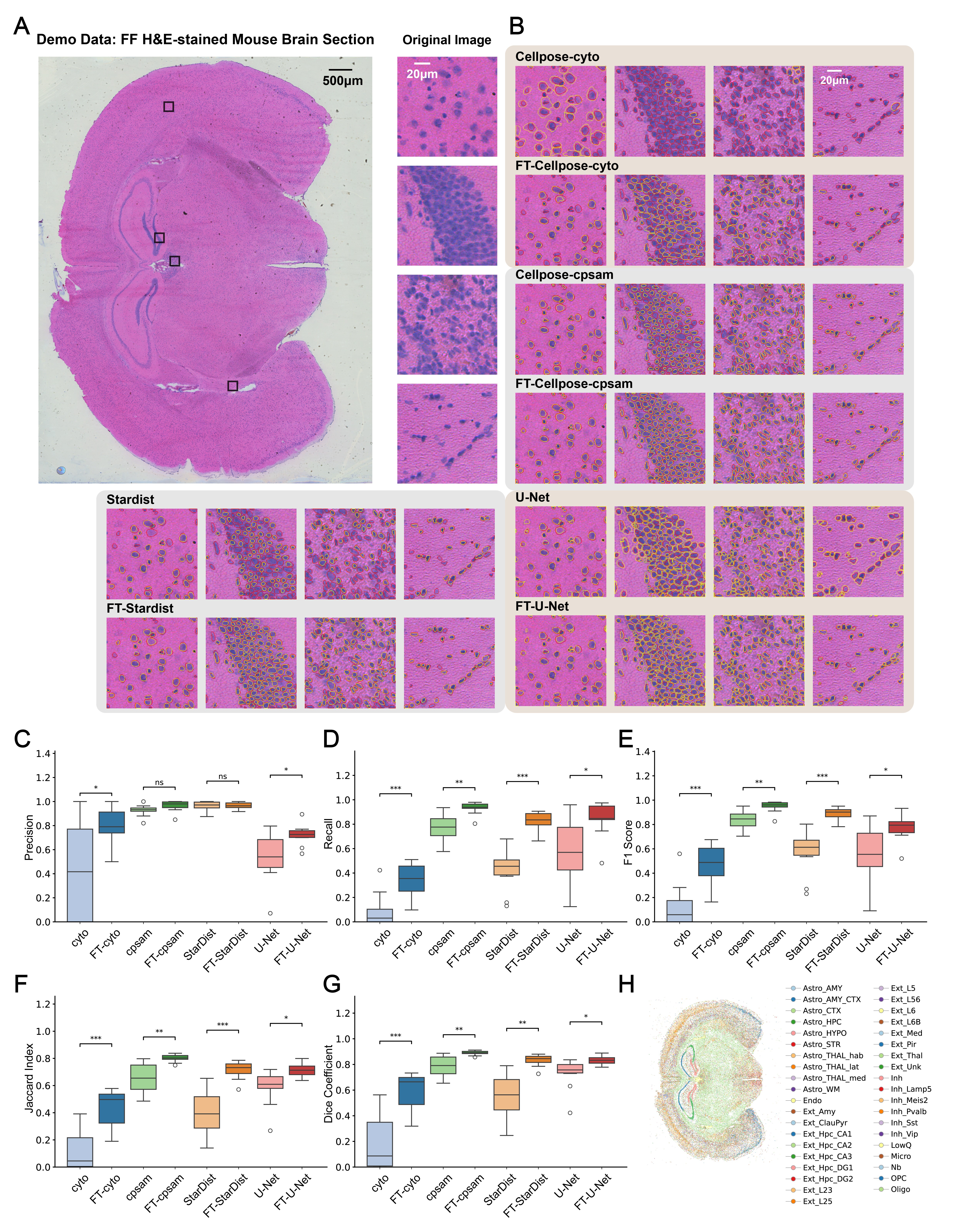

Supplement: Supplementary_Figure1_bbaf718 [file supplementary_figure1_bbaf718.jpeg]

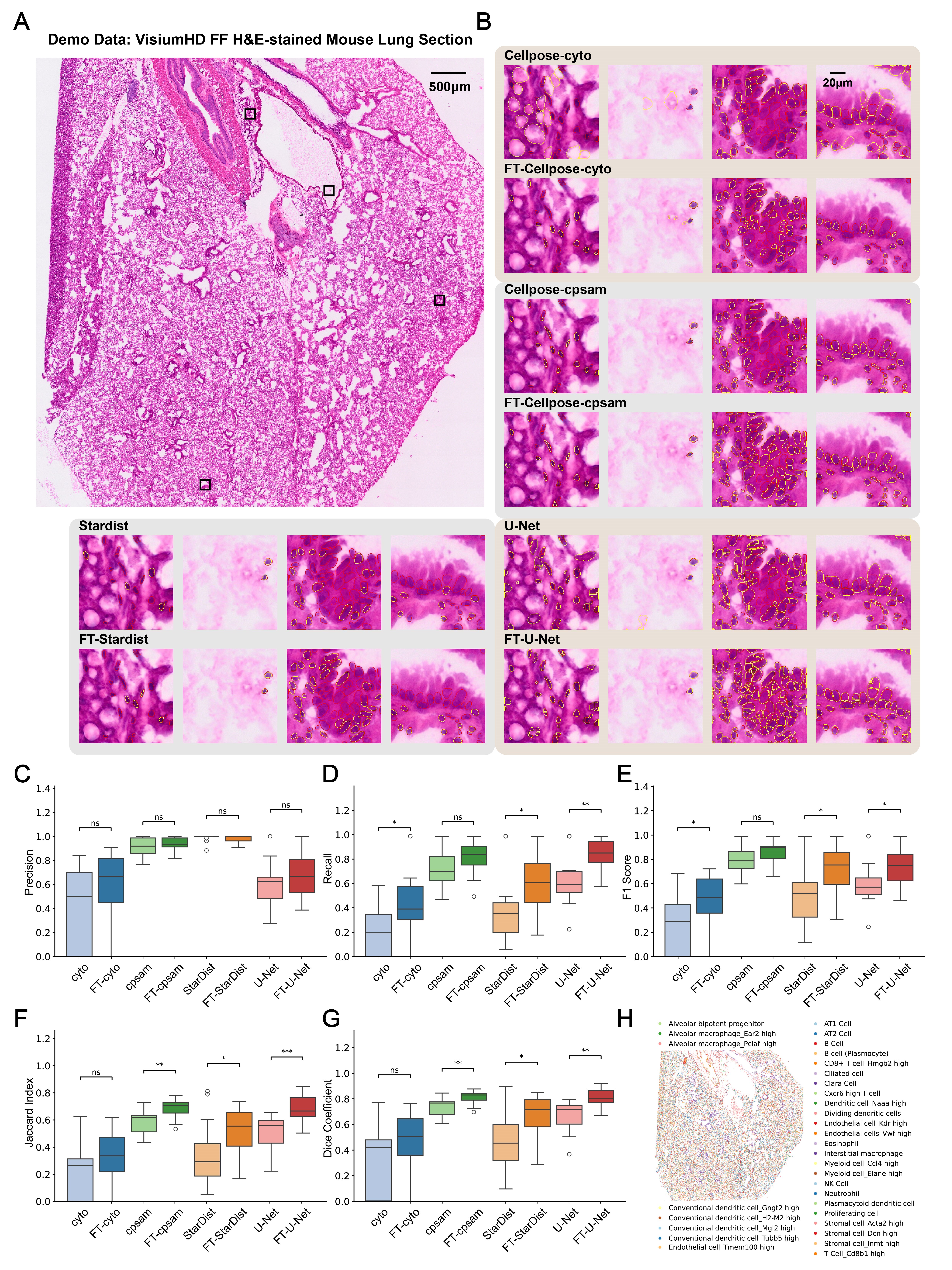

Supplement: Supplementary_Figure2_bbaf718 [file supplementary_figure2_bbaf718.jpeg]

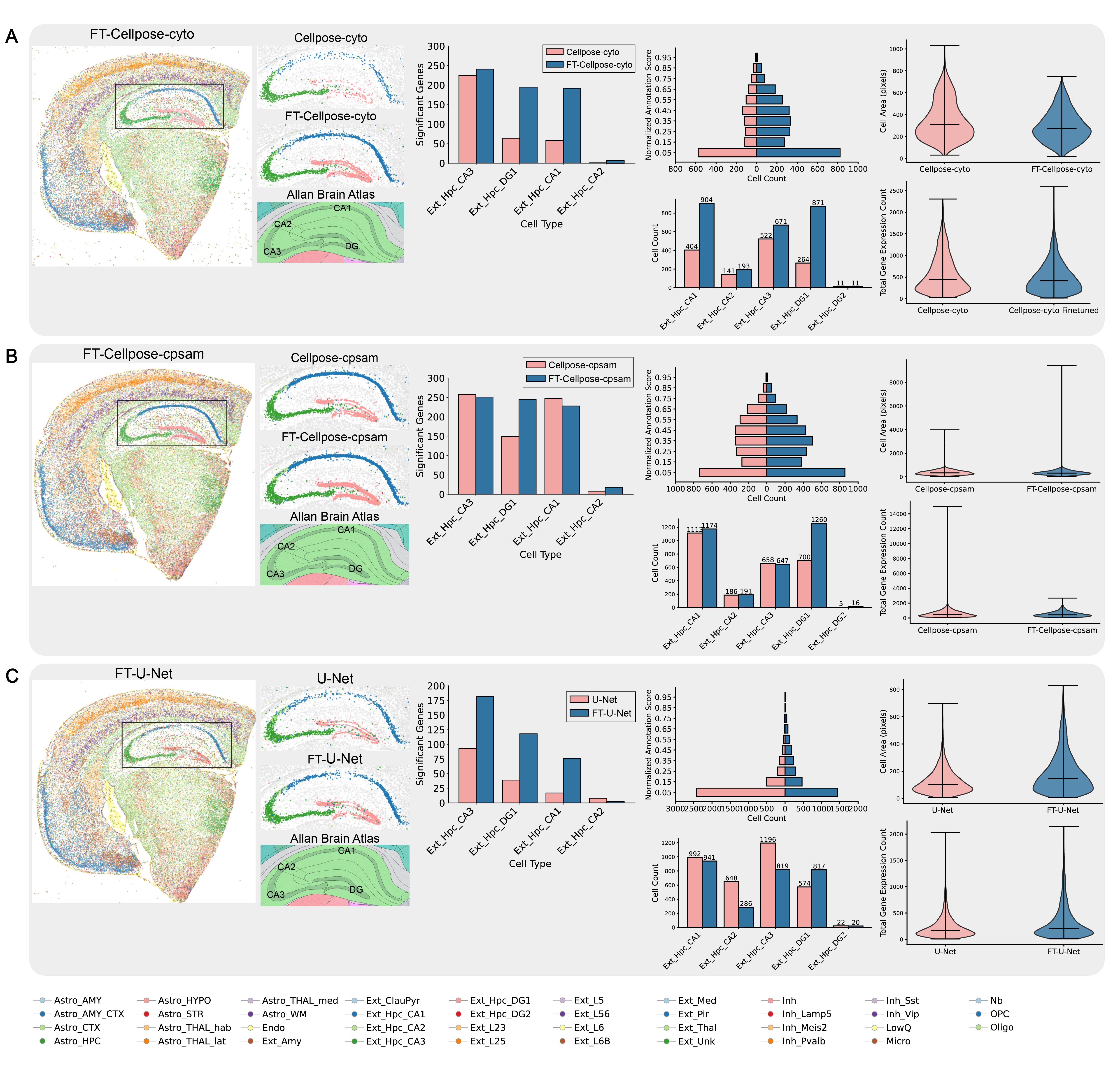

Supplement: Supplementary_Figure3_bbaf718 [file supplementary_figure3_bbaf718.jpeg]
