# Supplementary material for: CSRefiner: a lightweight framework for fine-tuning cell segmentation models with small datasets
Source: Brief Bioinform. 2026 Jan 13;27(1):bbaf718. doi: 10.1093/bib/bbaf718 (PMC12796817; doi:10.1093/bib/bbaf718)
Supplement: Supplementary_Figure_Legend_bbaf718 [file supplementary_figure_legend_bbaf718.docx]

### Supplementary Figure Legend

**Supplementary Figure 1. Performance of pre-trained and fine-tuned segmentation models on FF H&E-stained mouse brain section.** (A) FF H&E-stained mouse brain slice images (image resolution: 0.5 µm/pixel) with 4 example regions. (B) Segmentation results of four pre-trained models (Cellpose-cyto, Cellpose-cpsam, StarDist-2D_versatile_he, and U-Net) and their fine-tuned versions (prefixed with “FT-”) on the 4 example regions, including both hippocampal and non-hippocampal regions. Red outlines denote manually annotated ground truth; yellow outlines denote model-predicted segmentation boundaries. (C–G) Quantitative evaluation of segmentation performance before and after fine-tuning across four representative models. Boxplots show improvements in (C) Precision, (D) Recall, (E) F1 score, (F) Jaccard Index, and (G) Dice Coefficient. The significance mark line and P value are added to the box plot. The number of “*” from 1 to 3 represents a P value less than 0.05, 0.01, and 0.001. (H) Spatial maps of cell type annotations generated by cell2location using cgef matrices from the fine-tuned cpsam model.

Alt text:

Multi-panel figure showing FF H&E-stained mouse brain section images, segmentation results, quantitative performance evaluation, and spatial cell type maps. Example microscopy regions are shown alongside segmentation overlays comparing pre-trained and fine-tuned models, with ground truth and predicted boundaries. Boxplots display segmentation metrics before and after fine-tuning, and a spatial map illustrates cell type annotations generated from fine-tuned model outputs.

**Supplementary Figure 2. Performance of pre-trained and fine-tuned segmentation models on FF H&E-stained mouse brain section.** (A) FF H&E-stained mouse lung slice images (image resolution: 0.26 µm/pixel) with 4 example regions. (B) Segmentation results of four pre-trained models (Cellpose-cyto, Cellpose-cpsam, StarDist-2D_versatile_he, and U-Net) and their fine-tuned versions (prefixed with “FT-”) on the 4 example regions. Red outlines denote manually annotated ground truth; yellow outlines denote model-predicted segmentation boundaries. (C–G) Quantitative evaluation of segmentation performance before and after fine-tuning across four representative models. Boxplots show improvements in (C) Precision, (D) Recall, (E) F1 score, (F) Jaccard index, and (G) Dice coefficient. The significance mark line and P value are added to the box plot. The number of “*” from 1 to 3 represents a P value less than 0.05, 0.01, and 0.001. (H) Spatial maps of cell type annotations generated by cell2location using cgef matrices from the fine-tuned cpsam model.

Alt text:

Multi-panel figure showing FF H&E-stained mouse lung section images, segmentation results, quantitative performance evaluation, and spatial cell type maps. Microscopy images from multiple regions are presented with segmentation overlays comparing pre-trained and fine-tuned models. Boxplots summarize segmentation metrics before and after fine-tuning, and a spatial map displays cell type annotations generated from fine-tuned model outputs.

**Supplementary Figure 3. Fine-tuning improves biological interpretation reliability across Cellpose and U-Net models.** (A) Cellpose-cyto results; (B) Cellpose-cpsam results; (C) U-Net results. For each model, the figure contains: Spatial cell-type annotation maps generated by cell2location using cgef matrices derived from the fine-tuned model; Visualization of hippocampal subregion segmentation and annotated cells before and after fine-tuning, compared with the corresponding regions in the Allen Brain Atlas; Comparison of the number of marker genes significantly enriched in each hippocampal cell subtype before and after fine-tuning; Distribution of normalized annotation scores for all annotated cells before and after fine-tuning; Distribution of cell areas before and after fine-tuning; Comparison of the number of annotated cells per subtype in the hippocampus before and after fine-tuning; Distribution of detected gene counts per cell before and after fine-tuning.

Alt text:

Multi-panel figure comparing results from Cellpose-cyto, Cellpose-cpsam, and U-Net models. Panels include spatial cell type annotation maps, visualizations of hippocampal segmentation and annotated cells before and after fine-tuning, distributions of annotation scores and cell areas, comparisons of cell subtype counts and detected gene numbers, and summaries of marker gene enrichment across hippocampal cell subtypes.
