# Supplementary material for: CSRefiner: a lightweight framework for fine-tuning cell segmentation models with small datasets
Source: Brief Bioinform. 2026 Jan 13;27(1):bbaf718. doi: 10.1093/bib/bbaf718 (PMC12796817; doi:10.1093/bib/bbaf718)
Supplement: Supplementary_Table_1_bbaf718 [file supplementary_table_1_bbaf718.docx]

**Supplementary Table 1. Details of Training&Test Sets**

| Stereo-seq FFPE DAPI Mouse Brain (Z96512T5) | Training Set | Number of Patches | 20 |
| --- | --- | --- | --- |
|  |  | Patch Size (pixels) | 256×256 |
|  |  | Average Annotated Cells per Patch | 72.5 |
|  |  | Spatial Selection of Patches | Hippocampus (13) + non-hippocampus (7) |
|  | Test Set | Number of Patches | 6 |
|  |  | Patch Size (pixels) | 256×256 |
|  |  | Average Annotated Cells per Patch | 72.5 |
|  |  | Spatial Selection of Patches | Hippocampus (3) + non-hippocampus (3) |
| Stereo-seq FF H&E Mouse Brain (X96165X3) | Training Set | Number of Patches | 10 |
|  |  | Patch Size (pixels) | 256×256 |
|  |  | Average Annotated Cells per Patch | 92.4 |
|  |  | Spatial Selection of Patches | Hippocampus (4) + non-hippocampus (6) |
| VisiumHD FF H&E Mouse Lung | Training Set | Number of Patches | 15 |
|  |  | Patch Size (pixels) | 256×256 |
|  |  | Average Annotated Cells per Patch | 50.4 |
|  |  | Spatial Selection of Patches | Alveoli (8) + non-alveolar (7) |

Note: The specific location coordinates of each patch in WSI can be found in the filename, named in the format SN_length_width_x-axis_y-axis. The file download path can be found in Data availability.
