# Supplementary material for: CSRefiner: a lightweight framework for fine-tuning cell segmentation models with small datasets
Source: Brief Bioinform. 2026 Jan 13;27(1):bbaf718. doi: 10.1093/bib/bbaf718 (PMC12796817; doi:10.1093/bib/bbaf718)
Supplement: Supplementary_Table_2_bbaf718 [file supplementary_table_2_bbaf718.docx]

**Supplementary Table 2. Fine-tuning Hyperparameters**

| Model | Cellpose-cyto | Cellpose-cpsam | StarDist | U-Net |
| --- | --- | --- | --- | --- |
| Optimizer | SGD | SGD | Adam | Adam |
| Learning Rate | 0.2 | 1e-5 | 3e-4 | 1e-4 |
| Learning Rate Decay | 1e-05 | 0.1 | ReduceLROnPlateau (factor=0.5, patience=40) | ReduceLROnPlateau (monitor='val_loss', factor=0.1, patience=10, min_delta=1e-4) |
| Batch Size | 8 | 1 | 4 | 6 |
| Epochs (FFPE DAPI Mouse Brain) | 100 | 100 | early stopping at epoch 5 | early stopping at epoch 174 |
| Epochs (FF H&E Mouse Brain) | 100 | 100 | early stopping at epoch 4 | early stopping at epoch 156 |
| Epochs (FF H&E Mouse Lung) | 100 | 100 | early stopping at epoch 4 | early stopping at epoch 212 |
| Frozen Layers | None | None | None | None |
